# Evaluating the utility of the HAS‐BLED bleeding‐estimator tool for transurethral resection of prostate

**DOI:** 10.1002/bco2.480

**Published:** 2025-01-13

**Authors:** Lu Yu Kuo, Zhong Li Titus Lim, Caitlin Letch, Joshua Silverman, Jason Jae Yeun Kim, Scott McClintock

**Affiliations:** ^1^ Department of Urology Gold Coast University Hostpial Southport Queensland Australia; ^2^ Department of Urology Princess Alexandra Hospital Woolloongabba Queensland Australia; ^3^ School of Medicine and Dentistry Griffith University Southport Queensland Australia

**Keywords:** bleeding, bleeding calculator, haematuria, HAS‐BLED, transurethral prostatectomy, TURP

## Abstract

**Objectives:**

To evaluate the utility of the HAS‐BLED bleeding risk‐estimation tool to predict for clinically significant postoperative haematuria in patients receiving transurethral resection of prostate (TURP).

**Patients and Methods:**

A single‐centre, retrospective cohort analysis of patients underwent TURP from April 2019 to December 2023 for treatment of symptomatic benign prostate hyperplasia. The primary objective was to evaluate reliability of HAS‐BLED score in predicting postoperative bleeding event. A focus sub‐analysis was performed on anticoagulated patient cohort. Each patient was categorised in to HASBLED low‐, moderate‐ and high‐risk group according to the preestablished estimator tool. Patients' demographics, clinical, pathological and operative details were collected. Events of clinically significant haematuria within 3 months postoperatively were captured. Cohort characteristics and outcome were analysed with two‐sided *t* test and ANOVA test. Further weight‐adjusted multivariable analysis and ROC curve was performed to evaluate the predictive value of HAS‐BLED score.

**Results:**

Our analysis showed that patients assigned as high‐risk by HAS‐BLED were at 2.17‐times higher chance of developing clinically significant haematuria compared to the low‐risk patients. The risk for high‐risk patient was 18.5% (95%CI 11.7–25.3%) and 8.5% (95%CI 4.6–12.4%) for low‐risk patients. Moderate‐risk did not demonstrate any significant difference relative to the low‐risk group. Sub‐analysis of 113 patients receiving long‐term anticoagulation accentuates the utility of the tool. The risk of haematuria for high‐risk patient was 32.7% (95%CI 20.7–44.7%), moderate‐risk patient was 28.7% (95%CI 17.0–40.3%), and low‐risk patient was 9.7% (95%CI 4.2–15.2%). In this cohort, the risk of haematuria was 3.37 and 2.96 times higher in the high and moderate‐risk compared to the low‐risk group, respectively.

**Conclusion:**

This is the first study to validate a bleeding estimator tool for TURP patients. High HAS‐BLED score positively predicts clinically significant post‐TURP haematuria, particularly for patients receiving anticoagulation therapy.

## 1 INTRODUCTION

Benign prostatic hyperplasia (BPH) is prevalent amongst men above 50 years of age and requires treatment when patients develop clinically significant lower urinary tract symptoms (LUTS).^1^ Despite the development of various urological procedures for treatment of symptomatic BPH, TURP remains the predominant and readily available surgical option.^2^, ^3^ Familiar to TURP and other transurethral prostate, a degree of gross haematuria after the procedure is expected within 2 to 3 weeks postoperatively.^4^ It is considered a high bleeding‐risk procedure with considerable morbidity attributable to postoperative bleeding. It has been demonstrated that postoperative haematuria is the leading cause for emergency representations and re‐admissions up to 3 months postsurgery in a population base study and placed significant strain on the health care system.^5^

Preoperative multi‐disciplinary assessment and patient optimisation are imperative to minimise complications.^6^ Several studies have shown general risk factors such as older age, increased co‐morbidities and receiving long‐term anticoagulation to be independent predictors of developing significant haematuria post‐TURP.^5^, ^7^, ^8^ However, at present, there is no validated bleeding risk‐estimation tool specific for TURP to guide clinicians in delineating postoperative bleeding risk as part of preoperative assessment.

The need for a risk‐estimation tool is further accentuated by current epidemiological trajectories. Clinicians are expected to encounter more advanced‐age patients with increasingly complex co‐morbidities requiring surgical intervention for LUTS as the prevalence of symptomatic BPH is expected to rise.^9^ The increasing prevalence of atrial fibrillation (AF) also translate into increasing number of individuals receiving long‐term anticoagulation. The current AF prevalence of 46.3 million individuals globally is expected to triple in the coming decades with up to 70% of this population being anticoagulated.^10^, ^11^

There are several well‐established bleeding risk‐estimation tools, such as the HAS‐BLED, HEMORR_2_HAGES and ATRIA scoring systems.^12^, ^13^, ^14^ These scoring systems are predominantly designed to predict probability of bleeding events when commencing patients on anticoagulation. HAS‐BLED (hypertension, abnormal renal/liver function, stroke, bleeding history or predisposition, labile international normalised ratio, elderly [age >65 years], drugs/alcohol) risk estimation tool was selected for validation its apparent superiority in predicting clinically relevant bleeding and its simplicity to use.^15^ We further stratified the HAS‐BLED scoring system to low‐risk, moderate‐risk and high‐risk categories for the purpose of this study (Table 1).

**TABLE 1:** Breakdown of the scoring criteria for the HAS‐BLED bleeding estimator tool. Further categorisation of HAS‐BLED score to low‐, moderate‐ and high‐risk groups.

HAS‐BLED risk factor (points)	Scoring criteria
**H**ypertension (1)	Systolic blood pressure > 160 mmHg
**A**bnormal	
Renal function (1)	Creatinine >200 μmol/L, dialysis or renal transplant
Liver function (1)	Chronic hepatic disease (i.e. cirrhosis) or bilirubin ×2 normal, AST/ALT/ALP ×3 normal
**S**troke history (1)	History of stroke at time of entry
**B**leeding history/predisposition (1)	Prior major bleeding—defined as any bleeding required hospitalisation, haemoglobin drop of 20 mg/dL or transfusion requirement
**L**abile INR (1)	Deranged INR at entry
**E**lderly age (1)	Age of ≥ 65 years
**D**rug	
Antiplatelet/NSAID (1)	Use of antiplatelet or NSAID therapy
Alcohol excess (1)	≥ 8 Standard drinks per week
**Risk stratification**	**HAS‐BLED score**
Low risk	0–1
Moderate risk	2
High risk	≥ 3

The known risk factors for post‐TURP haematuria, which are advanced age, increased co‐morbidities and receiving anticoagulation, are expected to be more frequently encountered in the future with current epidemiological trajectories. We set out to validate the HAS‐BLED risk‐estimation tool to develop a quantitative measure of bleeding risk assessment for TURP patients. We preference HAS‐BLED score for its apparent superiority and ease to use. Our objective is to evaluate the utility of HAS‐BLED score for predicting clinically significant postoperative haematuria for TURP patients. The primary outcome is to assess correlation between patient's HAS‐BLED score and risk of bleeding complications.

## 2 METHODS

### 2.1 Study population and data collection

We conducted a 5‐year retrospective cohort study of all adult men that underwent elective TURP between 2019 to December 2023 at a single tertiary centre in Gold Coast, Australia. All patients received preoperative multi‐disciplinary assessment from the urologist, anaesthetist, pharmacist and other relevant specialty, if indicated. Routine preoperative blood test was performed including full blood count, electrolyte, liver function, renal function and coagulation profile. State‐wide electronic medical record system was used to retrieve patient data. Each patient was assigned a HAS‐BLED score according to their age, documented medical history and results of preoperative blood test (Table 1). Several additional variables including patient's demographics and perioperative details were collected. Demographics including age, ethnicity and body mass index (BMI) were recorded. Relevant preoperative details including urinary catheter dependence, treatment with 5‐alpha reductase (5‐ARI) medical therapy, prostate specific antigen (PSA) level, preexisting clinically significant prostate cancer and prostate size estimate. Patient co‐morbidity level was assigned by anaesthetists with the American Society of Anaesthesiologist (ASA) physical status classification. Anticoagulation and antiplatelet therapy were recorded as part of preoperative pharmacist review. Details including indication, perioperative withholding duration and bridging therapy were recorded. Operative details were captured, including duration of resection, energy delivery method (monopolar/bipolar) and tissue resected.

Outcome was measured in development of clinically significant haematuria. This was defined as acute representation to hospital for haematuria. The haematuria is further defined as requiring clinical assessment and observation, clot retention requiring catheterisation and bladder irrigation, and significant haematuria requiring return to theatre for cystoscopic bladder washout. All representations were captured through the state‐wide electronic medical record system for public hospitals. All patients had routine outpatient follow‐up at 8–12 weeks postoperative. Assessment of prolonged haematuria, defined as haematuria greater than 2 weeks, would be assessed at this point.

### 2.2 Statistical analysis

Stata Statistical Software: release 18 (Stata Corp., College Station, TX, USA) was used to perform data analysis. Each patient's HAS‐BLED score was stratified as low risk (HAS‐BLED, 0–1), moderate risk (HAS‐BLED, 2) and high risk (HAS‐BLED, ≥ 3). Comparison was made between each risk groups. One‐way analysis of variance (ANOVA) was performed to compare continuous variables. Chi‐square test was used for categorical variables. The primary analysis included the total cohort, and a secondary sub‐analysis was performed for patients receiving long‐term oral anticoagulation only. Subsequent weight adjusted model with inverse‐probability‐weighted statistical analysis were performed. The weight adjusted model was built on independent variables with statistically significant finding amongst the stratified HAS‐BLED risk groups using univariate logistic regression analysis. The weight adjusted model was applied for analysis for the full patient cohort and sub‐analysis with the anticoagulated cohort. A receiver‐operative characteristic (ROC) curve was used to assess the predictability of the HAS‐BLED risk groups.

## 3 RESULTS

### 3.1 Cohort summary

**FIGURE 1:**
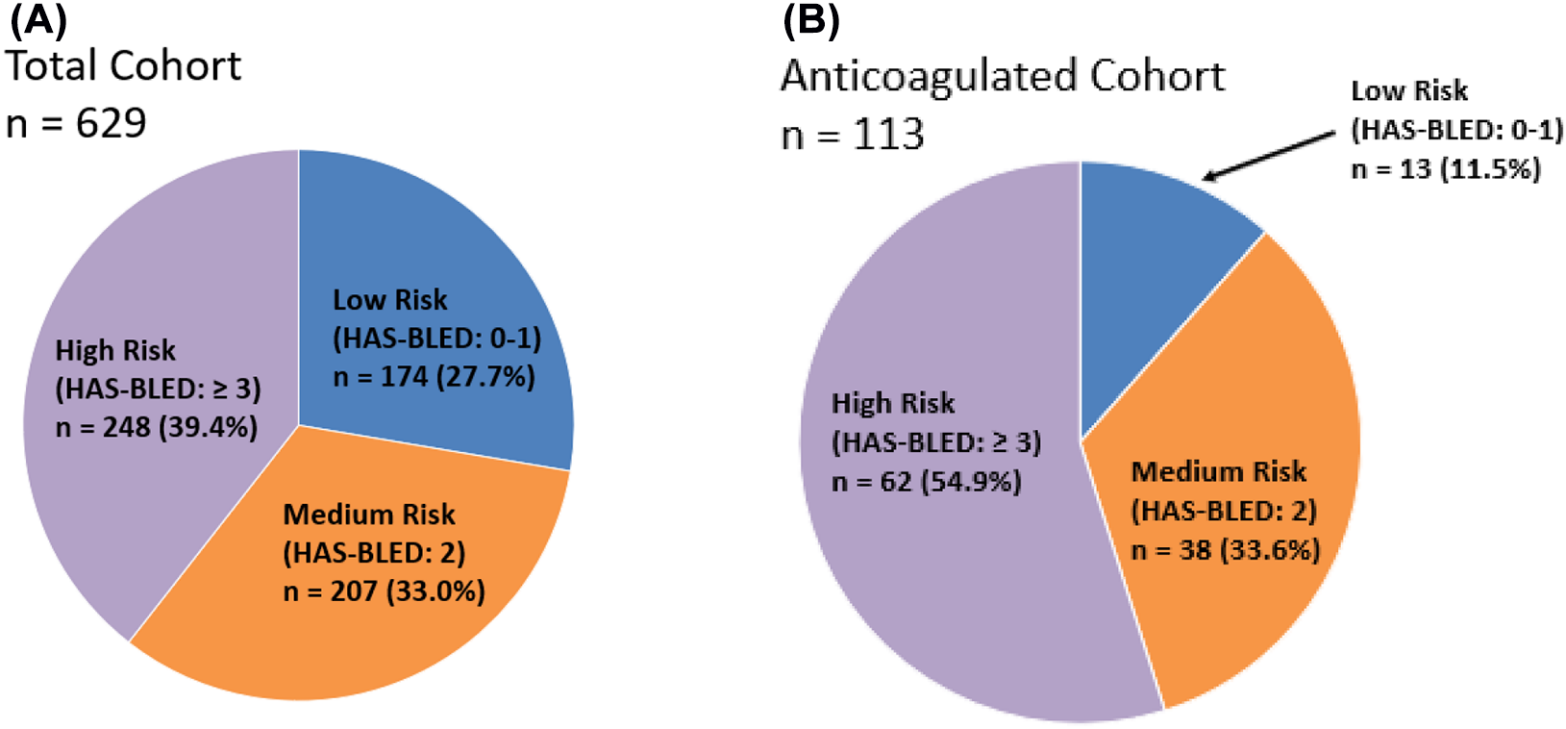
Cohort distribution of patients according to HAS‐BLED risk group from low‐, moderate‐ and high‐risk group. (A) Demonstration for the total cohort *n* = 629 patients. (B) Demonstration of the anticoagulated cohort *n* = 113 patients for subanalysis.

There are 629 patients in the 5‐year cohort with 113 (17.9%) anticoagulated patients for sub‐analysis (Figure 1). In the total cohort, the HAS‐BLED score distribution was as follows: 174 (27.7%) in the low‐risk group (HAS‐BLED score 0–1), 207 (33.0%) moderate‐risk (HAS‐BLED score 2) and 248 (39.4%) high‐risk group (HAS‐BLED score ≥ 3). In the anticoagulated cohort, the HAS‐BLED score distribution was noted to skew predominantly towards moderate and high‐risk groups with 13 (11.5%) low‐risk, 38 (33.6%) moderate‐risk and 62 (54.9%) high‐risk patients. Two‐sided *t* test comparison of the two groups demonstrated that the anticoagulated cohort were significantly older in age, higher in BMI, more likely to be treated with 5‐ARI, had preoperative urinary tract infection (UTI) and with higher ASA score, reflecting more significant medical co‐morbidities (Table S1). It was worth noting that anticoagulation status was not accounted for with the HAS‐BLED estimation tool and did not influence patient's risk score.

### 3.2 Primary outcome (total cohort analysis)

Cohort characteristics were compared between HAS‐BLED low‐risk to high‐risk patients (Table 2). There was a significant increase in age as patient risk categories progressed from low to high‐risk category. Patient with high‐risk HAS‐BLED score were more likely to have lower haemoglobin levels at their baseline, higher incidence of preoperative UTI requiring treatment and more likely to be on long‐term anticoagulation. It was observed that 25% of patients within the high‐risk group were anticoagulated, and their anticoagulation therapy would be withheld for longer period prior to TURP compared to other groups. Patient co‐morbidities appeared to increase when comparing low‐risk to high‐risk patients. This was reflected from progressive increase percentages of patients with ASA score of 3 and 4 as HAS‐BLED score progress from moderate‐ to high‐risk. Several other independent preoperative and operative parameters did not appear to have any statistically significant difference amongst the three risk groups. However, it was worth noting that there were equal percentages of patients in each risk group receiving 5‐ARI therapy prior to TURP, with 52% of patients in low‐risk group and 53% in moderate‐ and high‐risk groups.

**TABLE 2:** Cohort characteristics and outcome comparison of the total cohort according to HAS‐BLED risk group—low, moderate and high risk.

Total cohort, *n* = 629	HAS‐BLED risk total	Low (*n* = 174)	Moderate (*n* = 207)	High (*n* = 248)	*p*
Preoperative parameter					
Mean age	72.9 [47–95]	68.0	74.1	75.4	<0.001
Mean body mass index	28.0 [18–53]	27.7	27.8	28.5	0.21
Mean PSA (ng/mL)	5.9	5.7	6.2	5.9	0.89
Mean prostate volume (ml)	67.8	70.7	69.9	64.0	0.09
Mean haemoglobin (g/L)	137.8	142.3	137.7	134.7	<0.001
Bladder catheter dependent	308 (49%)	79 (45%)	100 (49%)	129 (52%)	0.36
Urinary tract infection (treated)	306 (49%)	68 (39%)	106 (51%)	132 (53%)	0.01
Treatment with 5‐ARI	91 (52%)	91 (52%)	110 (53%)	131 (53%)	0.97
Anticoagulated	113 (18%)	13 (7%)	38 (18%)	62 (25%)	<0.001
Operative parameter					
ASA score					
1	21 (3%)	16 (9%)	1 (1%)	4 (2%)	<0.001
2	316 (50%)	126 (72%)	107 (52%)	83 (34%)	
3	273 (43%)	32 (18%)	96 (46%)	145 (59%)	
4	20 (3%)	1 (1%)	3(1%)	16 (6.5%)	
Mean days of withhold AC (if anticoagulated)	4.5 (*n* = 112)	8.5 (*n* = 13)	4 (*n* = 38)	3.9 (*n* = 61)	0.02
Mean resection size (g)	19.1 9 [0.5–130]	20.5	18.6	18.4	0.37
Mean operative time (min)	47.2 [7–160]	50.4	45.7	46.2	0.21
Energy delivery methods					
Monopolar	306 (49%)	81 (46%)	94 (45%)	131 (53%)	0.23
Bipolar	324 (51%)	94 (54%)	113 (55%)	117 (47%)	0.23
Mean days irrigation post‐op	1.5 [1–17]	1.4	1.4	1.6	0.01
Complications					
**Haematuria related representation**	**82 (13%)**	**13 (7%)**	**19 (9%)**	**50 (20%)**	**<0.001**
Conservative	33 (5.2%)	7 (4%)	7 (3.4%)	19 (7.7%)	
Re‐catheterise for irrigation	42 (6.7%)	5 (2.9%)	10 (4.8%)	27 (10.9%)	
Cystoscopy washout	7 (1.1%)	1 (0.6%)	2 (1.0%)	4 (1.6%)	
Blood transfusion requirement	10 (1.6%)	5 (2.9%)	3 (1.5%)	2 (0.8%)	0.25
Prolonged haematuria	29 (4.6%)	6 (3.6%)	9 (4.5%)	14 (5.8%)	0.58
Mean days of prolonged haematuria	36.3	18	43	40	0.02
Thromboembolic related event					
Stroke	5 (0.8%)	0 (0%)	2 (1%)	3 (1.2%)	0.36
Acute coronary syndrome	2 (0.3%)	2 (1.1%)	0	0	
PE/DVT	2 (0.3%)	2 (1.1%)	0	0	
All‐cause mortality	3 (0.5%)	1 (0.6%)	0	2 (0.8%)	0.45
TURP related death	1 (0.15%)	0	0	1 (0.4%)	

Within 90‐day postoperative follow‐up, there were 82 (13%) acute hospital representations for haematuria related complication (Table 2). Of the patient represented, 33 (5.2%) were managed conservatively after clinical assessment and blood works. Forty‐two (6.7%) required re‐catheterisation and admission for bladder irrigation. Seven (1.1%) patients underwent cystoscopy washout due to significant clot retention. There were 10 (1.6%) patients who developed transfusion requirements postoperatively.

Patient with increased HAS‐BLED score had significantly higher risk of representing to hospital with clinically significant haematuria. In our analysis, we observed a progressive increase percentages of acute bleeding events from low‐risk to high‐risk groups. Only 13 low‐risk and 19 moderate‐risk patients developed clinically significant haematuria whereas 50 high‐risk patients developed such complications (7% vs. 9% vs. 20%, *p* < 0.001). It was worth noting that there were higher portion of high‐risk patients required re‐catheterisation for bladder irrigation (2.9% vs. 4.8% vs. 10.9%, *p* < 0.001) and cystoscopy washout for heavy clot retention (0.6% vs. 1.0% vs. 1.6%, *p* < 0.001). Despite having higher‐risk of bleeding and lower baseline haemoglobin level, we did not observe an increase in transfusion requirement for the HAS‐BLED high‐risk patients. However, developing transfusion requirement was a rare event, and our study may not have enough power to further assess this.

There was no significant difference between proportion of patients reporting prolonged haematuria of more than 14 days amongst the low, moderate and high HAS‐BLED risk groups (3.6% vs. 4.5% vs. 5.8%, *p* = 0.58). On further analysis for all patients reported prolonged haematuria, it appeared that duration of haematuria increased progressively from low to high‐risk patients for patients who reported prolonged haematuria (mean days 18, 43, 40, *p* = 0.02).

In addition to haematuria related complications, there were three death and nine thromboembolic events within 90 days of follow‐up. Only one of the death was related to the surgery from a postoperative stroke. Of all the non‐haematuria‐related complications, there was no statistically significant difference amongst the HAS‐BLED risk groups. It was to note that these were rare complications and would require larger cohort to be adequately assess.

### 3.3 Secondary outcome (anticoagulated cohort sub‐analysis)

A focus analysis was performed on the sub‐cohort with 113 patients receiving long‐term anticoagulation (Table 3). There were no significant differences in preoperative parameters amongst the low, moderate and high‐risk groups except for their age. It was observed that high‐risk patients appeared to have their anticoagulant withheld preoperatively for the shortest period compared to the low‐risk patient (mean days withholding 3.9 vs. 8.5 days, *p* = 0.02).

**TABLE 3:** Cohort characteristics and outcome comparison of the anticoagulated cohort according to HAS‐BLED risk group—low, moderate and high risk.

Total, *n* = 113	HAS‐BLED risk	Low (*n* = 13)	Moderate (*n* = 38)	High (*n* = 62)	*p*
Preoperative parameter					
Mean age	76.2 [53–95]	70.8	75.9	77.4	0.01
Mean body mass index	29.2 [18–48]	28.5	27.8	30.2	0.17
Mean PSA (ng/mL)	5.6	4.5	5.0	6.4	0.75
Mean prostate volume (mL)	67.8	63.2	71.8	66.6	0.71
Mean haemoglobin (g/L)	136	142	136	135	0.52
Bladder catheter dependent	58 (51%)	7 (54%)	19 (50%)	32 (52%)	0.97
Urinary tract infection (treated)	66 (58%)	6 (46%)	22 (58%)	38 (61%)	0.60
Treatment with 5‐ARI	69 (61%)	9 (69%)	21 (55%)	39 (63%)	0.61
Anticoagulated	113 (100%)	13 (100%)	38 (100%)	62 (100%)	
Operative parameter					
ASA score					
1	0 (0%)	0 (0%)	0 (0%)	0 (0%)	0.41
2	31 (27%)	4 (31%)	14 (37%)	13 (21%)	
3	71 (63%)	8 (61%)	22 (58%)	41 (66%)	
4	11 (10%)	1 (8%)	2 (5%)	8 (13%)	
Mean days of withhold AC (if anticoagulated)	4.5	8.5	4	3.9	0.02
Mean resection size (g)	20.0 [0.9–130]	21.0	19.8	19.9	0.98
Mean operative time (min)	47.2 [13–136]	50.2	45.0	49.1	0.76
Energy delivery methods					
Monopolar	47 (42%)	3 (23%)	17 (45%)	44 (27%)	0.35
Bipolar	66 (66%)	10 (77%)	21 (55%)	35 (56%)	0.35
Mean days irrigation post‐op	1.6 [1–17]	1.4	1.5	1.7	0.71
Complications					
**Haematuria related representation**	**31 (27%)**	**2 (15%)**	**8 (21%)**	**21 (34%)**	0.22
Conservative	12 (11%)	0 (0%)	4 (11%)	8 (13%)	
Re‐catheterise for irrigation	18 (16%)	2 (15%)	4 (11%)	12 (19%)	
Cystoscopy washout	1 (1%)	0 (0%)	0 (0%)	1 (2%)	
Blood transfusion requirement	5 (4.4%)	0 (0%)	1 (2.6%)	4 (6.5%)	0.47
Prolonged haematuria	21 (19.1%)	3 (23%)	8 (21.6%)	10 (16.8%)	0.77
Mean days of prolonged haematuria	42	16	43	48	0.02

In the unadjusted analysis, there was no difference in risk of developing clinically significant haematuria amongst the three risk groups in the anticoagulated cohort. We observed an increase percentages of haematuria acute representation progressively from the HAS‐BLED low, moderate to high‐risk groups (15% vs. 21% vs. 34%, *p* = 0.22), but this finding was not statistically significant. Limited to size of the cohort, our analysis did not have enough power to further define any differences in severity of bleeding. Further to this, there were no statistical significance in developing transfusion requirement (0%, 2.6%, 6.5%, *p* = 0.47). Again, we speculate that this may be attributed to the small number in this cohort. For the patients who reported prolonged haematuria, we observed that the number of days of haematuria increased as with HAS‐BLED risk group from low to high (mean days 16 vs. 43 vs. 48, *p* = 0.02).

### 3.4 Weight‐adjusted analysis and ROC curve

Further analyses were performed for the total and the anticoagulated cohort to consolidate the predictability of HAS‐BLED risk groups to clinically significant haematuria of all severity. Univariate logistic regression analysis for 12 independent variables were carried out on both cohorts to identify variables with statistically significant finding on haematuria representation and HAS‐BLED score risk stratification (Table S2). These significant variables were carried forward to build a weight‐adjusted model for analysis to assess predictability of HAS‐BLED risk groups to clinically significant haematuria. For the total cohort, variables significant amongst HAS‐BLED risk groups were patient age, preoperative haemoglobin, preoperative UTI, ASA score and anticoagulated status. Variables significant for clinically significant haematuria were patient BMI, catheter dependence, PSA level, 5‐ARI therapy, resection size, days of irrigation and ASA score. PSA level was excluded from the weight‐adjusted model as one‐quarter of the patients did not have a recorded preoperative PSA, and inclusion could introduce significant unintended bias on our analysis. For the anticoagulated cohort, variable significant amongst the HAS‐BLED risk groups were patient age only. Variables significant for clinically significant haematuria were catheter dependence, ASA score and received bridging therapy prior to TURP.

Our weight‐adjusted analysis demonstrated positive correlation between HAS‐BLED risk groups and risk of developing clinically significant haematuria (Table 4). In the total cohort, patients with HAS‐BLED low and moderate risk were at similar risk of representation with clinically significant haematuria and high‐risk patient is at 2.17 times higher risk compared to the low‐risk group (low‐risk 8.5%, *p* < 0.001; moderate‐risk 9.8%, *p* = 0.638; high‐risk 18.5%, *p* < 0.001). For the anticoagulated cohort, patient with HAS‐BLED moderate risk was at 2.96 times higher risk compared to low risk (low‐risk 9.7%, *p* < 0.001; moderate risk 28.7%, *p* = 0.001). HAS‐BLED high‐risk patients was at 3.37 times risk compared to low risk (low risk 9.7%, *p =* <0.001; high‐risk 32.7%, *p* < 0.001). It was worth noting that the positive predictive value of HAS‐BLED score were accentuated in the anticoagulated cohort and was able to observe significant difference amongst low‐, moderate‐ and high‐risk groups.

**TABLE 4:** Weight‐adjusted analysis for total and anticoagulated cohort.

	Clinically significant bleeding		
	Risk of bleeding %	95% *C. I*	*p*
Total cohort (*n* = 629)			
HAS‐BLED low risk	8.5%	4.6–12.4%	<0.001
HAS‐BLED moderate risk	9.8%	4.3–15.6%	0.636
HAS‐BLED high risk	18.5%	11.7–25.3%	0.004
Risk ratio			
(Moderate/low risk)	1.15		
(High/low risk)	2.17		
Anticoagulated cohort (*n* = 113)			
HAS‐BLED low risk	9.7%	4.2–15.2%	<0.001
HAS‐BLED moderate risk	28.7%	17–40.3%	0.001
HAS‐BLED high risk	32.7%	20.7–44.7%	<0.001
Risk ratio			
(Moderate/low risk)	2.96		
(High/low risk)	3.37		

**FIGURE 2:**
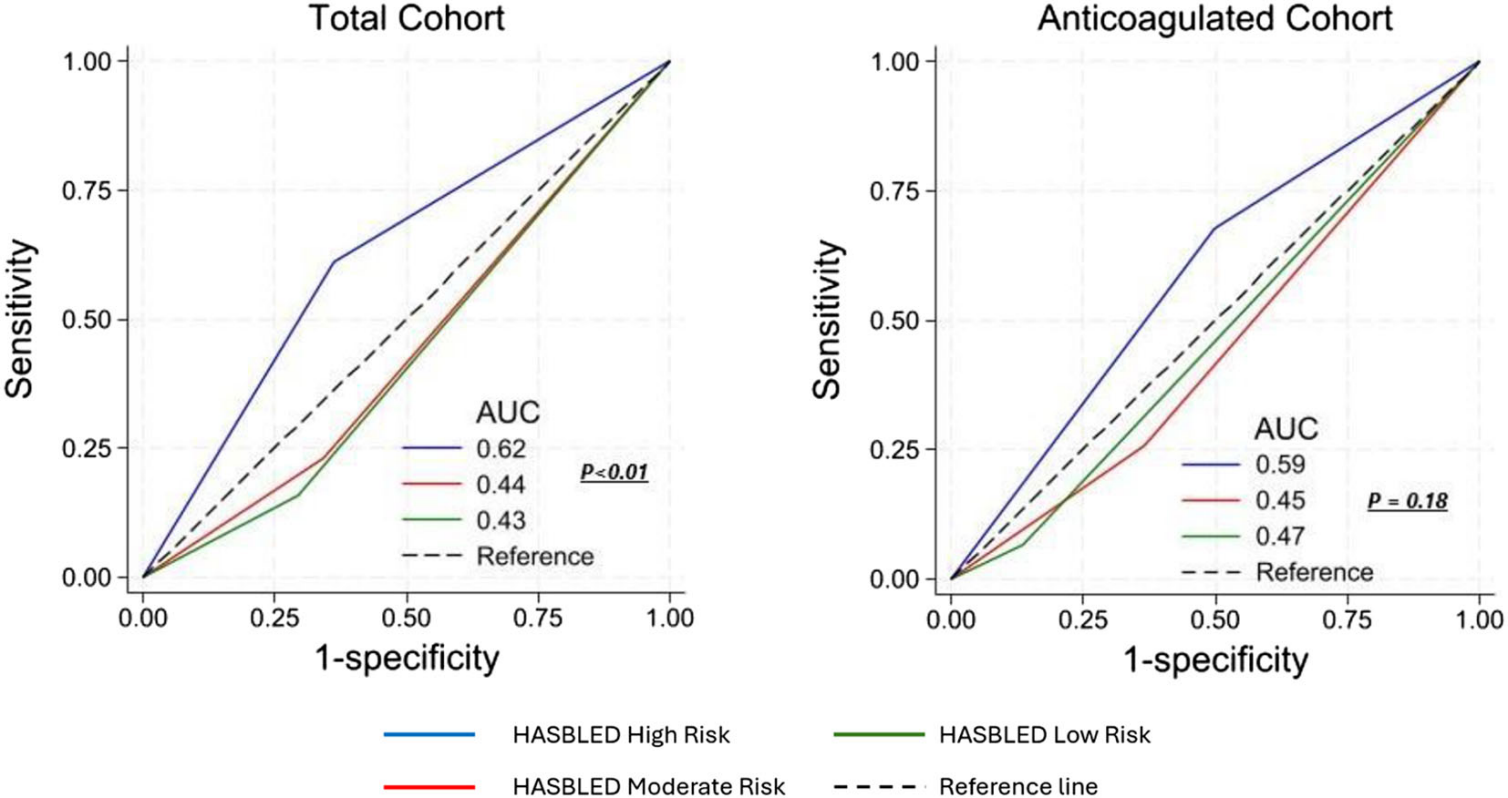
Receiver‐operative characteristic (ROC) curve of low‐, moderate‐ and high‐risk HASBLED groups for the total cohort and anticoagulated cohort. In the total cohort, the sensitivity of high‐risk categorisation for clinically significant bleeding was better compared low‐ and moderate‐risk (AUC 062 vs. 0.44 vs. 0.43, *p* < 0.01). Similar pattern was observed for the anticoagulated cohort, but the finding was statistically insignificant.

Similar finding as above have been demonstrated in our ROC analyses (Figure 2). On ROC analysis for the total cohort, HASBLED high‐risk category demonstrated significant predictive value for clinically significant haematuria (AUR 0.62, *p* < 0.01). On the contrary, HASBLED low‐ and moderate‐risk categories demonstrated negative predictive value (AUR 0.44, 0.43, respectively, *p* < 0.01). Similar finding was demonstrated in the ROC sub‐analysis for the anticoagulated cohort, but the finding was statistically insignificant.

## 4 DISCUSSION

To our knowledge, this is the first study to validate a risk‐estimation tool to offer clinician a quantitative bleeding‐risk assessment for patients undergoing TURP for BPH. We observed a significant percentage of patients categorised as HAS‐BLED high risk (score ≥ 3), 39.4% in the total cohort. This number increased further to 55% in the anticoagulated cohort. In our weight‐adjusted analysis, substantial numbers of high‐risk patients would develop clinically significant haematuria, from 1 in 5 for all patients to 1 in 3 if patients receive long‐term anticoagulation. It was worth noting that the high‐risk patients who developed clinically significant haematuria accounted for majority of all the haematuria complications, 61% and 68% for the total cohort and the anticoagulated cohort, respectively.

The above finding reflects that the HAS‐BLED score provides good guidance with identifying patients who are prone to developing postoperative haematuria. To reciprocate the high‐risk status, patients should receive trial of maximal medical therapy for symptomatic BPH prior to offering TURP.^16^ If surgical intervention is indicated, clinicians should consider implementing strategies perioperatively to reduce development of bleeding‐related events. Adequate preoperative assessment and optimisation is critical in reducing TURP‐related complication, and it has been demonstrated that an urgent TURP without appropriate multidisciplinary preoperative assessment led to increased risk of developing haematuria and acute urinary retention.^5^ Patient should be considered to commence on 5‐ARI as it has been demonstrated to reduce intraoperative and postoperative bleeding complications.^17^ Intraoperatively, clinicians could consider administration of tranexamic acid as it has been shown to significantly reduce intraoperative and immediate postoperative bleeding.^18^, ^19^ However, it is important to note that use of tranexamic acid has not been demonstrated to reduce the risk of delayed haematuria.

In our analysis, increased aged, increased ASA score and lower baseline haemoglobin level were associated with higher HAS‐BLED risk categories. These risk factors were similar from known TURP complications risk factors which consistently demonstrated older age and increased co‐morbidities (measured as ASA score in our cohort) were associated increased hospital representations with haematuria and acute urinary retentions.^5^, ^7^, ^20^ We also expected a higher HAS‐BLED score for older patients and patients with more medical co‐morbidities, as these factors were accounted for in the HAS‐BLED formula. The observation for lower baseline haemoglobin could relate to older age and co‐morbid patients within the HAS‐BLED high‐risk group, as these patients are more likely to have age related chronic anaemia and underlying pathology such as chronic kidney disease, nutritional deficiency and constellation of other chronic pathology.^21^

In our cohort, approximately one in five patients were receiving long‐term anticoagulant. We observed that anticoagulated patients were accounted for higher percentages of patients in the higher HAS‐BLED risk categories (low 7%, moderate 18% and high 25%, *p* < 0.001). This was despite anticoagulation status itself did not contribute to the HAS‐BLED formula. This finding could be explained by other confounding factors that anticoagulated patients tend to be older in age and had more medical co‐morbidities (Table S1) as these factors were accounted for by the HAS‐BLED formula. It was also worth noting that there was a statistically significant difference for the duration of anticoagulation therapy being withheld preoperatively (mean days 8.5, 4 and 3.9 days for HAS‐BLED low, moderate and high risk, respectively). We speculate that this finding may be associated with HAS‐BLED high‐risk patients had apparent elevated risk of developing thromboembolic disease due to older age and increased co‐morbidities as these pathologies were known to create imbalance in coagulation physiology.^22^ Clinicians were hence more reluctant to withhold anticoagulation therapy for prolonged period.

We observed a universal distribution of patients receiving preoperative treatment with 5‐alpha reductase inhibitor (5‐ARI) amongst the HAS‐BLED risk groups. In the total cohort, about one in two patients would be treated with 5‐ARI, whereas in the anticoagulated cohort, approximately 55–69% of patients received 5‐ARI amongst the low‐, moderate‐ and high‐risk groups (69%, 55%, 63% respectively, *p* = 0.60). There is significant amount of evidence in current literature to suggest treatment with 5‐ARI for as short as 2 weeks preoperative could significantly reduce intraoperative bleeding and postoperative transfusion requirement.^23^, ^24^ In our analysis, the HAS‐BLED high‐risk group patient in the anticoagulated cohort was predicted to have 32.7% risk of developing clinically significant haematuria, yet only 63% of patients within this group were receiving preoperative 5‐ARI. It would be worth considering initiating these patients on 5‐ARI prior to surgery in hope to minimise bleeding complications. However, it is important to note that this would consider to be off‐label use for 5‐ARI as it is primarily approved for treatment of BPH. Additionally, some evidence had suggested that the haemostatic effect of finasteride may be more pronounced than dutasteride.^25^

The present study has limitations. Patient's HAS‐BLED score was assigned retrospectively. Such approach is prone to limitation of retrospective study in general with unintended selection bias from the data collection process and missing information from incomplete medical record. Further to this, scoring criteria for some components of the HAS‐BLED estimator‐tool are not pragmatic for clinical practice and open to user bias. Several risk factors such as patient's age, blood pressure and renal function are continuous variables. However, these factors have been used in the HAS‐BLED estimator in a dichotomous fashion. Some factors of the HASBLED score can fluctuate in time‐specific context, such as blood pressure, renal function and liver function from day‐to‐day basis. Due to fluctuating nature of these components, assessment of a person's assigned HAS‐BLED score could fluctuate. However, it is important to note that accounting time‐specific factors may be difficult and could over‐complicate the use of the bleeding estimator tool and making it less user‐friendly.

In conclusion, HAS‐BLED appears to be a useful bleed‐risk estimator to provide clinicians with a quantitative measure of bleeding risk for TURP patients. We have demonstrated patient in high‐risk category (HAS‐BLED score ≥ 3) is at overall 2.17 times risk of developing clinically significant haematuria postoperatively compared to patient with low to moderate risk (HAS‐BLED score 0–1 and 2). This risk is increased to 3.37 times if patient is receiving long‐term anticoagulation therapy. Majority of patients with clinically significant haematuria was categorised as HAS‐BLED high‐risk, we recommended maximising non‐surgical therapy prior to consideration of surgical management. If surgery is unavoidable, patient should be carefully consulted on risk of bleeding, and perioperative measure to optimise their bleeding risk is essential. Alternative therapy such as holmium laser enucleation of the prostate (HoLEP) could be considered, for its apparent less risk of postoperative haematuria.^26^, ^27^

## AUTHOR CONTRIBUTIONS

**Lu Yu Kuo:** Data collection, statistical analysis, and writing of manuscript. **Zhong Li Lim:** Data Collection and editing of manuscript. **Caitlin Letch:** Data collection and writing of manuscript. **Joshua Silverman and Jason Kim:** Data collection and editing of manuscript. **Scott McClintock:** Overall supervision and guidance of manuscript.

## CONFLICT OF INTEREST STATEMENT

The authors have not declared any conflict of interest.

## PREPRINT SERVER DISCLOSURE

This manuscript has not been published previously in print or preprint.

## Supporting information


**Table S1.** Comparisons of patient with or without oral anticoagulation.

**Table S2.** Univariate regression analysis of independent variables which influence acute bleeding representation and HAS‐BLED score in the total cohort. 12 different variables were analysed, only the variables with significant difference are demonstrated in this table.
