# Supplementary material for: Evaluating the utility of the HAS‐BLED bleeding‐estimator tool for transurethral resection of prostate
Source: BJUI Compass. 2025 Jan 13;6(1):e480. doi: 10.1002/bco2.480 (PMC11771491; doi:10.1002/bco2.480)
Supplement: Supplementary file 1 — Table S1. Comparisons of patient with or without oral anticoagulation. [file BCO2-6-e480-s002.docx]

| **Table S1.** Comparisons of patient with or without oral anticoagulation | | |  |  |  |
| --- | --- | --- | --- | --- | --- |
| Total Cohort | Total  (n = 629) | No AC  (n = 513) | | AC  (n = 113) | *P* |
| **Preoperative parameter** |  |  | |  |  |
| Mean age | 72.9 | 72.2 | | 76.2 | *<0.01* |
| Mean Body Mass Index | 28.0 | 27.8 | | 29.2 | *0.03* |
| Mean PSA (ng/ml) | 5.2 | 9.00 | | 5.64 | *0.07* |
| Mean Prostate Volume (ml) | 67.8 | 67.8 | | 67.8 | *0.99* |
| Mean Haemoglobin (g/L) | 137.8 | 138 | | 136 | *0.45* |
| Bladder Catheter Dependent | 308 (49.2%) | 250 (49%) | | 58 (51%) | *0.62* |
| Urinary Tract Infection (treated) | 306 (49%) | 240 (46%) | | 66 (58 %) | *0.02* |
| Treatment with 5-ARI + α-Blocker | 324 (51%) | 255 (49%) | | 69 (61 %) | *0.02* |
| **Operative parameter** |  |  | |  |  |
| ASA Score  1  2  3  4 | 21 (3.3%)  316 (50%)  273 (43%)  20 (3.2%) | 21 (4.1%)  285 (55%)  202 (39%)  9 (1.7%) | | 0 (0%)  31 (27%)  71 (63%)  11 (10%) | *<0.01* |
| Mean Resection Size (grams) | 19.0 [0.5 – 130] | 18.9 | | 20.0 | *0.59* |
| Mean operative time (minutes) | 47.2 [7-160] | 47.0 | | 47.8 | *0.79* |
| Energy Delivery Methods |  |  | |  |  |
| Monopolar | 306 (49%) | 259 (50%) | | 47 (41%) | *0.10* |
| Bipolar | 324 (51%) | 258 (50%) | | 66 (58%) | *0.10* |
