# Supplementary material for: Evaluating the utility of the HAS‐BLED bleeding‐estimator tool for transurethral resection of prostate
Source: BJUI Compass. 2025 Jan 13;6(1):e480. doi: 10.1002/bco2.480 (PMC11771491; doi:10.1002/bco2.480)
Supplement: Supplementary file 2 — Table S2. Univariate regression analysis of independent variables which influence acute bleeding representation and HAS‐BLED score in the total cohort. 12 different variables were analysed, only the variables with significant difference are demonstrated in this table. [file BCO2-6-e480-s001.docx]

| **Table S2.** Univariate regression analysis of independent variables which influence acute bleeding representation and HAS-BLED score in the total cohort. 12 different variables were analysed, only the variables with significant difference are demonstrated in this table. | | | | |
| --- | --- | --- | --- | --- |
| Parameters for analysis | Acute Bleeding representation | | HAS-BLED risk grops | |
|  | Odds ratio | *P* | Odds ratio | *P* |
| Age | 1.01 | *0.49* | 1.12 | *<0.01* |
| Body Mass Index | 1.06 | *0.01* | 1.02 | *0.35* |
| Serum Haemoglobin (g/L) | 1.00 | *0.51* | 0.98 | *<0.01* |
| IDC dependent | 0.55 | *0.02* | 1.23 | *0.24* |
| Pre-operative urinary tract infection (treated) | 1.01 | *0.98* | 1.71 | *<0.01* |
| PSA (ng/ml) | 0.95 | *0.03* | 1.01 | *0.68* |
| Treatment with 5-ARI | 1.87 | *0.01* | 1.04 | *0.83* |
| Resection size (g) | 1.02 | *<0.01* | 0.99 | *0.08* |
| ASA Score of 3-4 | 2.08 | *<0.01* | 4.82 | *<0.01* |
| Days of irrigation | 1.32 | *0.02* | 1.27 | *0.08* |
